# Impact of different models based on blood samples and images for bone marrow dosimetry after ^177^Lu-labeled somatostatin-receptor therapy

**DOI:** 10.1186/s40658-024-00615-5

**Published:** 2024-04-02

**Authors:** Delphine Vallot, Séverine Brillouet, Séléna Pondard, Lavinia Vija, Jean-Sébastien Texier, Lawrence Dierickx, Frédéric Courbon

**Affiliations:** Oncopole Claudius Regaud, Toulouse, France

**Keywords:** ^177^Lu-DOTATATE, Internal radiotherapy, Dosimetry, SPECT/CT, Bone marrow

## Abstract

**Background:**

Peptide receptor radionuclide therapy with ^177^Lu-DOTATATE is a recognized option for treating neuroendocrine tumors and has few toxicities, except for the kidneys and bone marrow. The bone marrow dose is generally derived from a SPECT/CT image-based method with four timepoints or from a blood-based method with up to 9 timepoints, but there is still no reference method. This retrospective single-center study on the same cohort of patients compared the calculated bone marrow dose administered with both methods using mono, bi- or tri-exponential models. For the image-based method, the dose was estimated using Planetdose© software. Pearson correlation coefficients were calculated. We also studied the impact of late timepoints for both methods.

**Results:**

The bone marrow dose was calculated for 131 treatments with the blood-based method and for 17 with the image-based method. In the former, the median absorbed dose was 15.3, 20.5 and 28.3 mGy/GBq with the mono-, bi- and tri-exponential model, respectively. With the image-based method, the median absorbed dose was 63.9, 41.9 and 60.8 with the mono-, bi- and tri-exponential model, respectively. Blood samples after 24h post-injection did not evidence any change in the absorbed bone marrow dose with the bi-exponential model. On the contrary, the 6-day post-injection timepoint was more informative with the image-based model.

**Conclusion:**

This study confirms that the estimated bone marrow dose is significantly lower with the blood-based method than with the image-based method. The blood-based method with a bi-exponential model proved particularly useful, without the need for blood samples after 24h post-injection. Nevertheless, this blood-based method is based on an assumption that needs to be more validated. The important difference between the two methods does not allow to determine the optimal one to estimate the true absorbed dose and further studies are necessary to compare with biological effects.

**Supplementary Information:**

The online version contains supplementary material available at 10.1186/s40658-024-00615-5.

## Introduction

Peptide receptor radionuclide therapy (PRRT) with ^177^Lu-DOTATATE or ^177^Lu-oxodotreotide, a somatostatin analog radiolabeled with ^177^Lu, is a recognized therapeutic option for metastatic or symptomatic patients with midgut WHO grade I-II neuroendocrine tumors. It has proven short- and long-term efficacy in terms of progression-free survival and symptom control even if the objective response rate was only 18%, as previously demonstrated by the NETTER-1 study [1]. The standard treatment consists in four intravenous injections of 7.4 GBq of ^177^Lu-DOTATATE administered every 8 weeks. This therapy is well tolerated as few toxicities have been reported for kidney and bone marrow [2, 3]. Nevertheless, severe hematotoxicity may occur. For example, the NETTER 01 study reported grade 3 or 4 thrombopenia (2%) and lymphopenia (9%), and myelodysplastic syndrome (MDS) can occur in about 2% and leukemia in about 1% of treated patients [4, 5]. Estimating the absorbed dose to the bone marrow to personalize and optimize treatment could possibly limit the hematological toxicity, which could be particularly challenging in the event of retreatment or further cytotoxic treatment. However, the methodology for estimating the absorbed dose in bone marrow is not standardized and the exact dose–effect relationship remain elusive. Significant but weak correlations between image-based estimates of the red-bone-marrow absorbed dose and hematological toxicity have been demonstrated [6]. Hagmarker et al. found a significant correlation between the absorbed dose in bone marrow and decreased platelet counts [7], whereas Garske-Roman et al*.* found that bone marrow dosimetry did not predict toxicity [8]. For Forrer et al*.* there is no correlation between red marrow absorbed dose and short-term acute hematological toxicity [9].

In the literature, the bone marrow dose is basically derived from an image-based method or from a blood-based method, with some crossover between both. Both have advantages and drawbacks. To calculate the time activity curve (TAC), both methods require several acquisitions or blood samples after each ^177^Lu-DOTATATE administration, which implies patient availability and compliance. In the imaging method, each acquisition lasts more than 40 min with possible motion artefacts. In both methods, the assumption is that the activity concentration in bone marrow is the same as in blood [9]. In the image-based method, it is assumed that the absorbed dose of a limited area, mostly the lumbar spine, represents the total bone marrow activity. No standard method yet exists, and Table 1 shows the published methods to calculate the bone marrow dose, acquisition or blood sampling timepoints, the model used, and the estimated absorbed dose in bone marrow. The threshold of the absorbed dose in bone marrow for severe hematological toxicity is still unclear and is dependent on the patient’s risk factors. It was initially set at 2 Gy based on ^131^I therapy data [10]. Considerable differences exist depending on the method used and few comparisons are available. Page et al*.* recommends using image-based dosimetry in clinical treatment for the red marrow dose as the blood-based method may underestimate it by a factor of 4 [11].

**Table 1:** Literature review of absorbed dose in bone marrow for ^177^Lu-PRRT

References	Author, year	Number of patients	Blood samples	Planar imaging	SPECT/CT imaging	Activity modeling	Bone marrow dosimetry	S factor	Bone marrow absorbed dose (Gy/GBq)
[12]	Kwekkeboom, 2001	5	10, 20, 40, 60 and 90 min and 2, 5 and 24 h p.i	4 h, and 1, 3, 10, 17 days p.i	–	–	Planar	–	0.07
[13]	Wehrmann, 2007	27	3, 10, 20,40 min and 1, 2, 4, 6, 20, 32, 44, 66, 70 h p.i	–	–	Bi or tri exponential	Blood	OLINDA	0.04 ± 0.02
[9]	Forrer, 2009	15	5 samples between 0 and 168 h p.i	3 images between 24 and 168 h p.i	–	–	Blood + planar	OLINDA	0.034 ± 0.03
[2]	Bodei, 2011	12	–	–	–	–	–	–	0.033
[14]	Jackson, 2013	17	–	–	4, 24 72 h p.i	Tri exponential	SPECT/CT	Monte-Carlo simulations	0.0334 ± 0.012
[15]	Sandström, 2013	200	0.5, 1, 2.5,4,8, 24 h p.i	24, 96, 168 h p.i	24, 96, 168 h p.i	Mono exponential	Blood + planar	RADAR	0.007—0.054
[4]	Bodei, 2015	10	–	–	–	–	–	–	0.03
[16]	Denoyer, 2015	11	–	–	4, 24, 72h p.i	–	SPECT/CT	OLINDA	0.0315
[17]	Bergsma, 2016	24	0,10,30,60,90,120, 360 1440 min p.i	24, 96, 168 h p.i	–	–	Blood + planar	OLINDA	0.067 ± 0.007
[18]	Svensson, 2016	46	–	2, 24, 48 168 h p.i	24 h p.i	Bi-exponential	Planar	RADAR	0.027 ± 0.007
[19]	Del Prete, 2017	22	–	–	4, 24 72 h p.i	Mono exponential	SPECT/CT	OLINDA	0.046 ± 0.033
[20]	Gosewich, 2018	5	30, 80 min and 24,48, 72 h p.i	24, 48, 72 h p.i	24, 48, 72 h p.i	Mono and bi exponential	Blood + SPECT/CT	RADAR	0.012 ± 0.003
[21]	Del Prete, 2019	34	–	–	4, 24 72 h p.i	Linear + mono exponential	SPECT/CT	OLINDA	0.035
[22]	Santoro, 2018	12	–	–	4, 24, 72 192 h p.i	Mono exponential	SPECT/CT	OLINDA	0.04 ± 0.02
[23]	Marin, 2018	47	30 min, 1, 4, 24 and 144–192 h p.i	–	4, 24, 144–192 h p.i	Bi-exponential	Blood + SPECT/CT	OLINDA	0.028 ± 0.01
[24]	Thakral, 2018	10	–	2, 24, 96 h p.i	–	Mono/bi exponential	Planar	OLINDA	0.017 ± 0.016
[25]	Chicheportiche, 2018	24	18, 20—25 h p.i	–	18, 25 h and 7 d p.i	Mono exponential	Blood + SPECT/CT	OLINDA	0.0096
[8]	Garske-Roman, 2018	200	0.5, 1, 2.5, 4, 8, 24 h p.i	1, 4, 7 d p.i	1, 4, 7 d p.i	Mono exponential	Blood + planar	-	0.018
[7]	Hagmarker, 2019	46	–	2, 24, 48, 168 h p.i	24 h p.i	Mono/bi-exponential	Planar + SPECT/CT	ICRP 133	0.016–0.287
[26]	Hallqvist, 2021	17	–	2, 24, 48, 168 h p.i	24 h p.i	Mono/bi-exponential	Planar + SPECT/CT	–	0.041
[27]	Carter, 2021	2	–	–	–	–	SPECT/CT	ICRP, Monte-Carlo simulations	0.043–0.13
[28]	Vergnaud, 2022	13	–	–	1, 24, 96 or 144 h p.i	Tri-exponential	SPECT/CT	Monte-Carlo simulations	0.04 ± 0.02

Recently, the EANM dosimetry committee published its recommendations for the dosimetry of ^177^Lu-labeled somatostatin-receptor and PSMA-targeting ligands [6]. For ^177^Lu-PRRT, the median value for the red marrow absorbed dose across all the studies is 50 mGy/GBq. The total-body time activity curve (TAC) is generally biphasic, as is the case for the blood TAC. The recommendation for dosimetry is to measure the activity concentration in the blood even if sequential planar or SPECT/CT whole-body imaging is also possible.

Another issue that hampers the use of dosimetry in general is the fact that late timepoints are crucial to correctly estimate the absorbed dose [6]. This late timepoint, which is usually somewhere between 144 and 168h post-injection, may be difficult to establish as it implies that the patient needs to come back to the institute. With the blood-based method, it is still not clear whether this late timepoint has any value.

To our knowledge, no publication on the two methods has compared the impact of different models for dosimetry with patients acting as their own control. The aim of this study was to evaluate and compare the impact of the mono-, bi- and tri-exponential models for the blood-based method and the image-based method. We evaluated and compared the impact of the late timepoints in both methods to estimate the bone marrow dose administered in ^177^Lu-DOTATATE therapy.

## Material and methods

### Patients

A retrospective study was conducted on 59 patients treated in our institution for neuroendocrine tumors (NET) with ^177^Lu-DOTATATE (Lutathera ®) between 2019 and 2021. In a standard study, patients received 4 cycles of ^177^Lu-DOTATATE at the recommended activity of 7400 MBq during a 30-min intravenous perfusion. The interval between each cycle was eight weeks. The study was declared to the Health Data Hub (number: F20230102114415).

### Blood sampling

Six to nine blood samples were collected at 0.5, 1, 2, 4, 8, 16, 24, 72 and 144 h post-injection in each patient. Blood was collected in the arm contralateral to that in which ^177^Lu-DOTATATE was injected. Blood samples were centrifuged at 1000xg for 10 min at ambient temperature. Radioactivity was measured in 1 ml aliquots of plasma using a WIZARD^2^™ 2480-0010 Gamma Counter (Perkin Elmer, MS, USA). A calibration curve with ^177^Lu-DOTATATE was established to normalize the data obtained with the gamma counter. Radioactivity-time data were expressed in MBq/L after correction for radioactive decay between the time of the sampling and the measurement according to the equation:

$${C(t)}_{corr}={C(t)}_{measured}\times {{\text{e}}}^{\left(\frac{-Ln\left(2\right)}{{T}_{\frac{1}{2},{177}_{Lu}}}({t}_{measured}-t)\right)}$$

where C(t)_corr_ is ^177^Lu-Dotatate concentration at time t corrected for radioactive decay, C(t)_measured_ is the ^177^Lu-Dotatate concentration measured at time t_measured_, T_1/2,_^177^_Lu_ is the half-time disintegration of ^177^Lu and T_0_ is the time of ^177^Lu-Dotatate injection [29].

### Image acquisition

Abdominal SPECT/CT acquisitions were planned at 4, 24, 96 and 144 h post-injection. The last timepoints were modified depending on weekend constraints. Images were acquired on a GE Discovery NM CT 670 system, which is composed of two 15.8 mm (5/8″) NaI (Tl) crystal detectors and a 40 × 54 cm axial FOV. Medium-energy general purpose (MEGP) collimators were used. All images were acquired with a 20% energy window around the main photopeak of ^177^Lu of 208 keV and a lower scatter window of 10% around 178 keV and an upper scatter window of 10% around 241 keV. Matrix size was 128 × 128 and 60 projections of 40 s were acquired over 360°. Images were reconstructed using the OSEM algorithm (5 iterations, 10 subsets) with correction of attenuation with low-dose CT images, scatter and resolution recovery.

### Dosimetry

For the blood-based method, the activity concentration was fitted by a mono-, bi- or tri- exponential curve to infinity to estimate the area under the curve (AUC) using an in-house Python program (Python 3.10, numpy and matplotlib libraries). The mass and self-dose S-value used to calculate the absorbed dose to the bone marrow were those of the EANM recommendations [6].

For the image-based method, dosimetry was performed using the PlanetDose® software from DOSIsoft. It used reconstructed images and allowed the full processing: organ-based rigid registration using CT images, rigid propagation of the structures, time integrated activity coefficient (TIAC) fitting (several fitting options) and organ absorbed dose (dose kernel or local deposition model with or without density correction) [30]. For bone marrow, the trabecular part of the vertebrae between L2 and L4 was delineated to estimate the absorbed dose [22, 31]. To reduce the error, the union of these three volumes of interest was considered in one structure. The mono-, bi- and tri-exponential models were used, as it commonly found in the literature and the absorbed dose was estimated by using the local deposition method with density correction.

For both methods, the used fitting functions had the following expression:

Mono-exponential: $$a*{e}^{-bx}$$

Bi-exponential: $$a*{e}^{-bx}+c*{e}^{-dx}$$

Tri-exponential: $$a*{e}^{-bx}+c*{e}^{-dx}+f*{e}^{-gx}$$

Where a, b, c, d, f and g were coefficients to determine.

The goodness of fit of the models was measured using R^2^.

To estimate the influence of the late timepoints for the blood-based method, we estimated the absorbed dose with only the timepoints before 24 h post-injection. For the image-based method, we kept only three timepoints by deleting the last one and compared the absorbed dose to the one calculated with all the timepoints.

### Statistics

To compare the different methods/models and the influence of the timepoints, Bland–Altman plots have been built.

## Results

Patient characteristics are shown in Table 2.

**Table 2:** Patient characteristics

	All patients (*n* = 59)
Gender, *n* (%)	
Male	38 (64.4)
Female	21 (35.6)
Age at first injection	
Median [min–max]	67 [26–83]
Site of primary tumor, *n* (%)	
Small intestine	51 (86.4)
Pancreas	5 (8.5)
Stomach	1 (1.7)
Unknown	2 (3.4)
Injected activity (MBq)	
Median [min–max]	7457.2 [3516–7989]

### Blood-based method

**Fig. 1:**
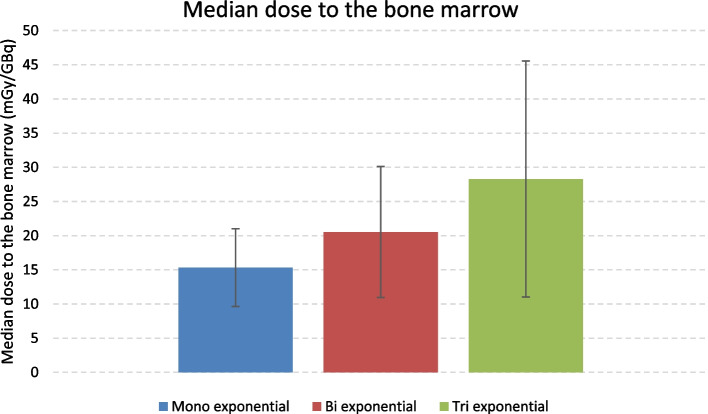
Median absorbed dose to bone marrow depending on model for blood-based method

As some patients received several ^177^Lu-DOTATATE infusions, the study took 131 treatments into account: 48 for the first infusion, 38 for the second, 26 for the third and 19 for the fourth. Before analyzing the 131 treatments, we investigated the possibility to pool all the data without differentiating the cycles. As the results were similar, we estimated that all the data could be analyzed without differentiating the cycles (see Additional file 1). Table 3 and Fig. 1 show the median absorbed dose to the bone marrow calculated with the mono-, bi- or tri-exponential model for the blood-based method.

**Table 3:** Median absorbed dose to bone marrow depending on model for blood-based method

	Blood-based method		
	Mono-exponential	Bi-exponential	Tri-exponential
Median absorbed dose to the bone marrow (mGy/GBq) [min–max]	15.3 [6.4–35.8]	20.5 [9.4–73.4]	28.3 [9.7–106.9]

For the evaluation of the fitting, the coefficient of determination (*R*^2^) was calculated. It was always higher for the tri-exponential model (median: 0.999, min: 0.849, max: 1) than for the bi-exponential model (median: 0.999, min: 0.849, max: 0.999). Its lower median value was for the mono-exponential model (median: 0.979, min: 0.849, max: 0.998).

For the first phase, the effective half-lives vary between 0.2 h and 5.8 h. For the second phase, they vary between 1.6 h and 34.6 h.

### Image-based method

Thirteen patients and seventeen treatments were studied for the influence of the model on the median absorbed dose to the bone marrow calculated with the image-based method (Table 4).

**Table 4:** Median absorbed dose to bone marrow depending on model for image-based method

	Image-based method		
	Mono-exponential	Bi-exponential	Tri-exponential
Median absorbed dose to bone marrow (mGy/GBq) [min–max]	63.9 [20.3–181.1]	41.9 [18.6–88.7]	60.8 [19.1–250.8]

The goodness of fit of the models was measured using R^2^. The median was 0.22 (min: −3.2, max: 0.36) for the mono-exponential model, 0.78 (min: 0.71, max: 0.99) for the bi-exponential model and 0.72 (min: −0.51, max: 0.99) for the tri-exponential model.

In four cases, the tri-exponential model gave non-physical results.

As the fitting is not good for the mono-exponential model, we did not consider this model in the rest of this article.

**Fig. 2:**
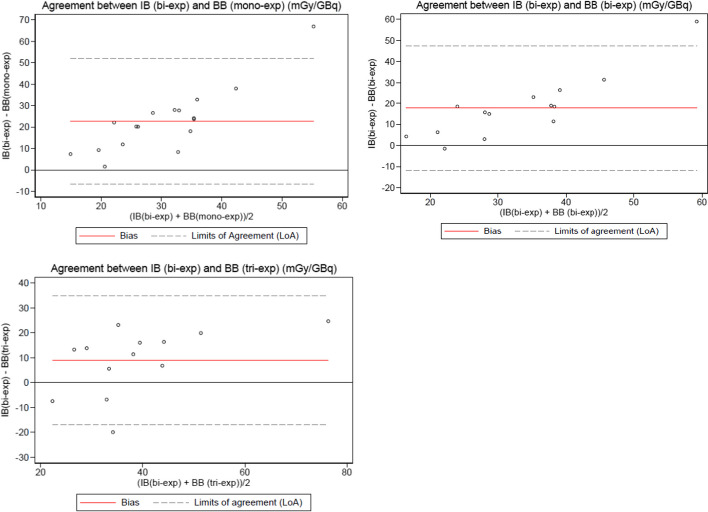
Bland–Altman plots showing the differences between the normalized absorbed bone marrow dose calculated with the bi-exponential model with the image-based (I.B) method and the mono, bi and tri exponential models with the blood-based (B.B) method

**Fig. 3:**
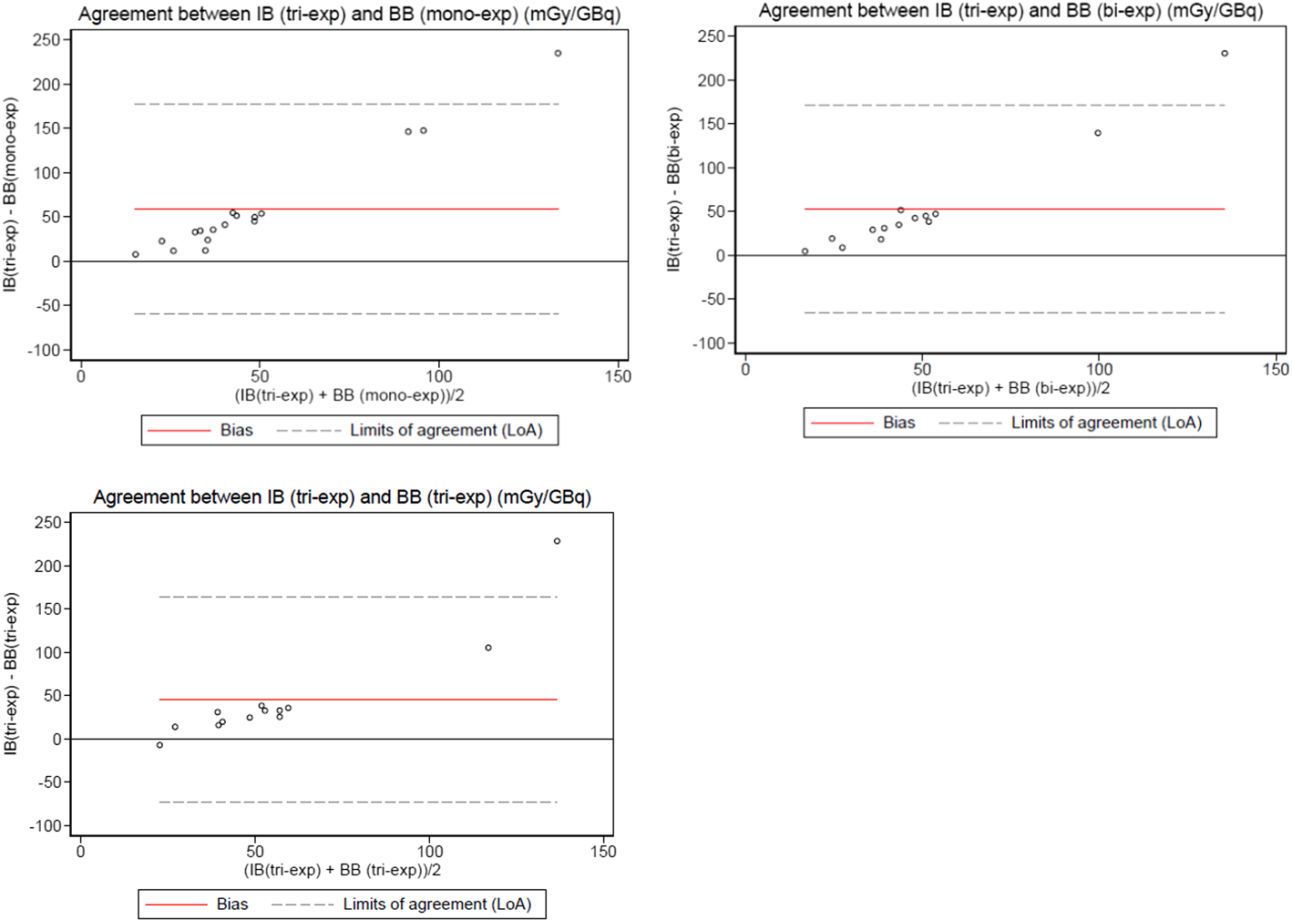
Bland–Altman plots showing the differences between the normalized absorbed bone marrow dose calculated with the tri-exponential model with the image-based (I.B) method and the mono, bi and tri exponential models with the blood-based (B.B) method

Differences between the two methods (image or blood) and the different exponential models are shown in Figs. 2 and 3 with the Bland–Altman plots.

It was not possible to find a relationship between the doses calculated with these two methods that would be usable for all the patients (Table 4). One example of the plots for a patient is given in Additional file 1: Figures S6 and S7.

### Influence of late timepoints

#### Blood-based method

To investigate the influence of the late timepoints in the blood-based method, we excluded the timepoints collected after 24 h in 45 treatments (Table 5).

**Table 5:** Influence of late timepoints with blood-based method

Model	Mono-exponential	Bi-exponential	Tri-exponential
Mean difference (%)	0	0.06	7.12
Minimal difference (%)	0	0	−16.5
Maximal difference (%)	0	1.12	48.95

**Fig. 4:**
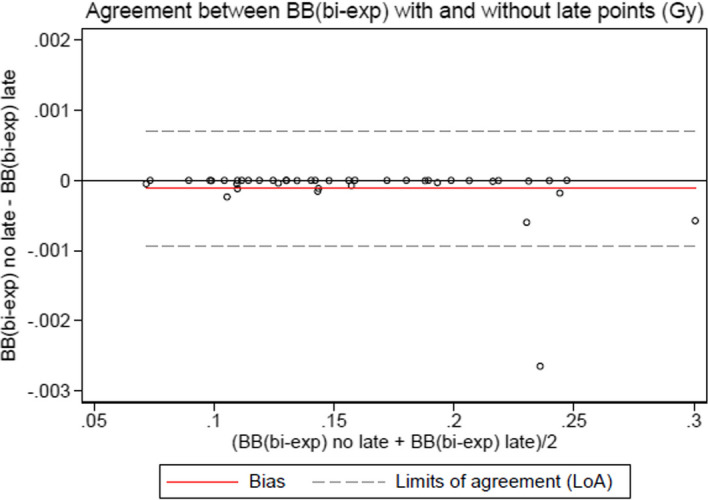
Bland–Altman plot for the bi-exponential model in the blood-based method comparing the absorbed bone marrow dose using late timepoints or not

**Fig. 5:**
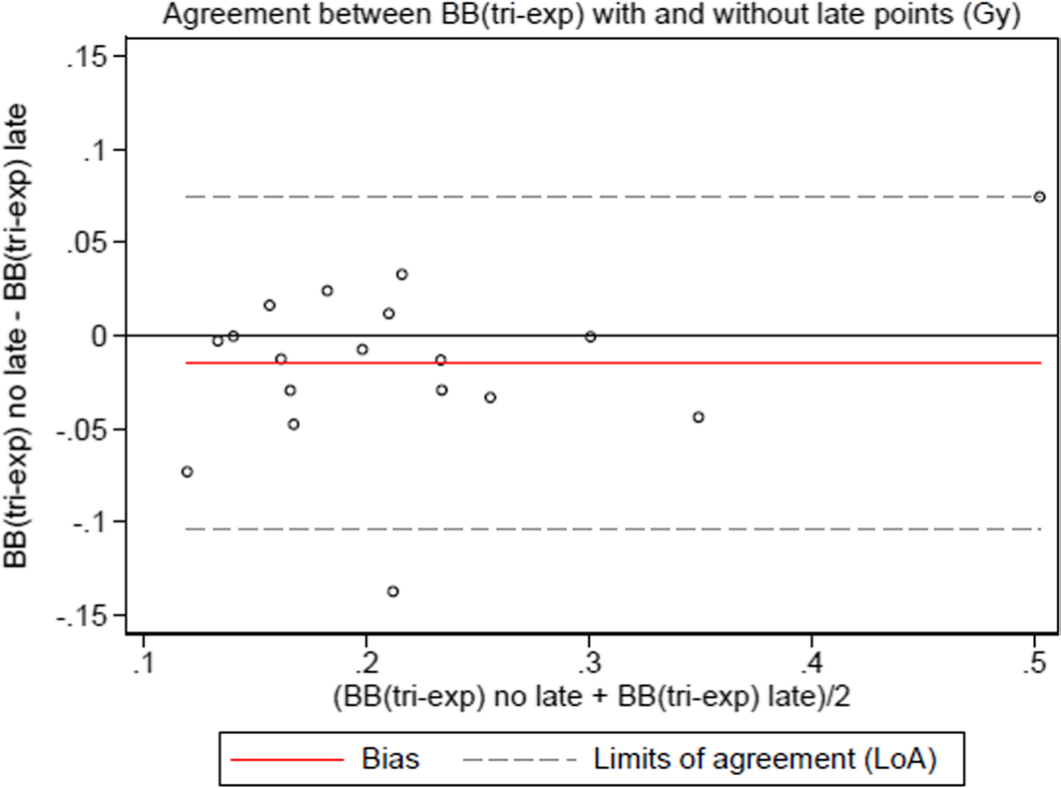
Bland–Altman plot for the tri-exponential model in the blood-based method comparing the absorbed bone marrow dose using late timepoints or not

The Bland–Altman plots to compare the influence of the late timepoints with the blood-based method are given in Fig. 4 for the bi-exponential model and Fig. 5 for the tri-exponential model. The plot for the mono-exponential model is not provided as it was not relevant (no difference).

#### Image-based method

To study the influence of the late timepoints in the image-based method, we excluded the fourth image in 11 treatments. The relative difference in the absorbed dose in bone marrow calculated with the bi-exponential model varied in most patients from −58.9 to 78%. In one patient, the dose varied from 0.32 to 1.3 Gy (311%). In another patient, it varied from 0.36 to 2.9 Gy (715%) by deleting the fourth timepoint.

## Discussion

In this retrospective study, we compared the image- and blood-based methods and used three models to calculate the absorbed dose in bone marrow after ^177^Lu-DOTATATE therapy. Three exponential models were studied for the blood-based method and the image-based method. The latter was limited by the small number of treatments.

The median absorbed dose ranged from 15.3 to 63.9 mGy/GBq depending on the method used, which is coherent with the median literature value of 50 mGy/GBq (range 2 to 150 mGy/GBq) [6] (see Table 1). With the blood-based method, the tri-exponential model gave a higher median absorbed dose than the bi-exponential model, which also gave a higher median dose than the mono-exponential model. The tri-exponential model was not always accurate and sometimes gave an infinite AUC, that is maybe due to the low number of points especially for the image-based method where we only have 4 points [32].

Like Page et al*.* [11] or Beykan et al. [33] who found that blood-based bone marrow absorbed doses were by a factor of three lower than image-based bone marrow absorbed doses, we observed that the absorbed dose in bone marrow calculated with the image-based method was systematically higher than that calculated with the blood-based method. This result was obtained whatever the model used. Hemmingsson et al. who found similar results, explain it by the presence of somatostatin-receptor type 2 on CD34-positive hematopoietic stem cells in the red marrow that causes a specific uptake in the red marrow through late elimination [34].

Lubberink et al. [35] showed a fast metabolism of ^177^Lu-DOTATATE: the fraction of intact Lutathera decreased rapidly during the first 24 h with the major part of radioactivity consisting of smaller metabolites. This finding could explain the much higher concentrations in bone marrow than in blood and so the much higher bone marrow-absorbed doses found for image-based than for blood-based dosimetry. Another element could be taken in consideration to explain these results: the transchelation competition of the DOTA chelator, used to link the radioisotope in ^177^Lu-DOTATATE, with the transferrin present in the blood perturbing the in vivo stability of ^177^Lu-DOTATATE [36]. What we know is that in addition to circulating blood, there is an expression of somatostatin receptors on lymphocytes and activated leukocyte subtypes involved in haematological toxicity [37, 38].

The lowest bias between the two methods was obtained when using a bi-exponential model with the image-based method, very likely because the blood TAC follows a bi-phasic pattern [39]. We also observed a loss of accuracy between the models when the doses increase. Unfortunately, we did not find a systematic relationship between the two methods because as Hemmingsson et al. concluded [34], it is highly patient-dependent. However, this result needs to be confirmed with more data.

Like Page et al. [11] or Hagmarker et al. [7], we observed that the way the absorbed dose was estimated had a great impact on the results. To the best of our knowledge, it is impossible to determine which value is the most reliable as the bone marrow dosimetry is the most challenging [40] and in ^177^Lu-DOTATATE therapy, it is not systematically linked to biological effects and toxicity prediction. Garske-Roman et al. [8] found that bone marrow dosimetry did not predict toxicity and Forrer et al. [9] that no conclusions can be drawn concerning the relationship between calculated bone marrow absorbed dose and risk of developing myelodysplastic syndrome. On the contrary, Hagmarker et al. [7] found a correlation between bone marrow absorbed dose and platelets count decreasing and Svensson et al. [18] a correlation between bone marrow absorbed dose and haematological toxicity.

The influence of the late timepoints seemed lesser with the blood-based method for points sampled after one day and using the mono or bi-exponential model, which is contrary to the literature [6]. On the other hand, a late timepoint is important when using the tri-exponential model as it tries to cover all the relevant part of the time activity curve. Late timepoints are required with the image-based method to obtain a relevant result.

Based on these data, it would now be interesting to use a population-based pharmacokinetic model to assess the absorbed dose in bone marrow after ^177^Lu-DOTATATE administration and to compare the findings with our results obtained by standard practices using image-based dosimetry and the blood-based method.

## Conclusion

By comparing different methods and models for the peptide receptor radionuclide therapy of gastroenteropancreatic neuroendocrine tumors using ^177^Lu-DOTATATE, we confirmed that the blood-based method estimates the bone marrow dose significantly lower than the image-based method. If the blood-based method is used, a bi-exponential model proved particularly useful as the estimated bone marrow dose with sampling time after 24h post-injection was not different from the one estimated without sampling time after 24 h post-injection. This method is more accessible as it takes less time for the patient and the gamma camera’s availability so the cost is reduced. Nevertheless, this blood-based method is based on an assumption that needs to be more validated.

As a result, the important difference between the two methods does not allow to determine the optimal one to estimate the true absorbed dose and further studies are necessary to compare with biological effects.

### Supplementary Information

**Additional file 1:** Comparison of the normalized absorbed dose to the bone marrow between the first cycle and the fourth one for 13 patients.

## Data Availability

The datasets used and/or analyzed during the current study are available from the corresponding author on reasonable request.

## Abbreviations

AUC: Area under the curve
MDS: Myelodysplastic syndrome
NET: Neuroendocrine tumors
PRRT: Peptide receptor radionuclide therapy
TAC: Time activity curve
