# Supplementary material for: Impact of different models based on blood samples and images for bone marrow dosimetry after 177Lu-labeled somatostatin-receptor therapy
Source: EJNMMI Phys. 2024 Apr 2;11:32. doi: 10.1186/s40658-024-00615-5 (PMC10987460; doi:10.1186/s40658-024-00615-5)
Supplement: Supplementary file 1 — Additional file 1: Comparison of the normalized absorbed dose to the bone marrow between the first cycle and the fourth one for 13 patients. [file 40658_2024_615_MOESM1_ESM.docx]

**Supplementary information**

We fixed one method (blood-based method) and one model (bi-exponential model) to evaluate the changes in the normalized absorbed dose to the bone marrow between the first (D_1_) and the fourth (D_4_) cycles. 13 patients had these two data. We calculated the ratio between the normalized absorbed dose to the bone marrow: D_4_/D_1_. The median and mean ratio was 0.989 and 0.993 respectively. Thanks to these results, we considered that we could make the analysis without differentiating the four cycles.

**Supplemental figures**

|  |  |
| --- | --- |

Figure 6: Measured data and bi/tri exponential model for image-based method for patient 6.

Figure 7: Measured data and bi/tri-exponential model for blood-based method for patient 6.
